# Social innovation for health-care delivery in Africa

**DOI:** 10.2471/BLT.17.020417

**Published:** 2017-04-01

## Abstract

Incentivizing health-care delivery can overcome barriers to health care in low-income countries. Claire Keeton reports.

Millie Balamu goes from door to door providing life-saving health care for about 200 households in the Wakiso district of Uganda.

Villagers call her *masawu* (“doctor” in the local Luganda language), but she is not a doctor, she is a community health worker.

Trained in 2011 and supported by a nongovernmental organization (NGO) called Living Goods, the mother-of-three always has tests and drugs with her to diagnose and treat malaria, diarrhoea and pneumonia – the major killers of children in Uganda. She uses her mobile phone to diagnose these diseases and register pregnant women for follow up.

“We’ve borrowed from the Avon lady model: our community health promoters are independent agents, not employees,” says Shaun Church, president of Living Goods based in San Francisco, the United States of America (USA). 

“Rather than selling make-up, they are selling affordable medicines and life-changing products and services.” 

The Living Goods’ project was launched in Uganda in 2007 and in Kenya in 2015. It is one of 23 projects in 43 countries that were selected by the Social Innovation in Health Initiative, out of a total of 170 nominated in 2015, as promising new ways to improve health-care delivery.

The Social Innovation in Health Initiative is a collaboration between the Special Programme for Research and Training in Tropical Diseases at the World Health Organization (WHO), the Bertha Centre for Social Entrepreneurship and Innovation at the University of Cape Town, the Skoll Centre for Social Entrepreneurship at Oxford University and the London School of Hygiene & Tropical Medicine.

For Dr Francois Bonnici, co-founder and director of the Bertha Centre, these projects propose novel solutions that are sustainable and can be scaled up in other places.

“These projects need to go beyond health provision to empower people and create jobs or some other social value for communities,” he says.

“These projects need to go beyond health provision to empower people and create jobs or some other social value for communities.”

Francois Bonnici

The concept of social innovation is taken from economics and business studies and refers to efforts to mobilize and incentivize communities.

In health, social innovation may refer to low-fee private delivery of health care, using mobile phone applications – such as the one Balamu uses to diagnose common childhood diseases – and other novel ways to make health-care delivery more accessible and affordable in low-income communities, Bonnici says.

According to a working paper presenting the results of a randomized controlled trial in Uganda of more than 8000 households, published in 2016 by the Center for Economic and Policy Research, the Living Goods project helped to reduce child mortality across those households by 27% between 2011 and 2013. 

Living Goods calculates that the approach costs less than US$ 2 per head of population served; costs that are covered by Living Goods. 

“The project was designed to tackle three problems undermining community health: finding volunteers in impoverished communities, lack of supervision and a poorly-stocked supply chain,” says Church.

“Each community worker carries what we call ‘a toolkit in a bag’. These are preventative and curative health products, including vitamin-fortified porridge, antimalarials and treatment for diarrhoea. They also sell household products such as stoves that run on clean energy sources, solar lamps and water filters.

“The demand for these items is high and the cost of getting them into the field is largely covered by the savings from buying them wholesale,” Church says.

Living Goods trains its community health workers, gives them an initial loan to buy the products at subsidized prices, and monitors and rewards their performance. The community health workers commit to working at least two hours a day, five days a week. Most of them work part time and make about US$ 10–15 per month from their sales mark up. The average monthly incomes in the villages where they work range from US$ 30–120.

Church says that, through partnerships with other NGOs in Myanmar, Uganda and Zambia, Living Goods hopes to reach 6.5 million people by the end of 2018.

Another of the 23 projects selected by the Social Innovation in Health Initiative is run by Kheth’Impilo, a South African NGO that addresses the severe shortage of pharmacists in South Africa.

The project seeks to increase the number of people ordering and dispensing essential medicines for some three million South Africans on antiretroviral treatment for HIV infection – of some seven million living with the virus – as well as for people living with diabetes, heart disease, hypertension and other chronic conditions.

The Kheth’Impilo project operates a nationally accredited training programme for high school graduates to become pharmacist assistants in four provinces of South Africa. Once qualified, they can work as pharmacists in the public and private sector under the direct supervision of pharmacists. 

These assistants can also perform certain duties in primary health-care clinics under the indirect supervision of a pharmacist, such as ordering medicines and dispensing them along with health advice. Indirect supervision means that the pharmacist is based at a nearby pharmacy and visits regularly to supervise the pharmacist assistants. 

The focus is on training people to work in communities where increasing the capacity to dispense medicines will have the most impact on health and on employment, in a country where one quarter of the 53 million population is unemployed.

“We draw on existing resources and try to strengthen communities by applying management principles, including motivating people, rigorous supervision and improved logistics,” says Dr Ashraf Grimwood, chief executive officer of Kheth’Impilo.

Under this apprenticeship model, pharmacist’s assistants spend four days a week in the clinic under supervision and one day in class. Students in this two-year programme have a 95% pass rate and a 99% employment rate.

“Once they have completed their training, the assistants can cover the majority of the qualified pharmacists’ work, while the pharmacist visits primary health-care facilities supervising pharmacy operations and keeping operations running smoothly,” says Lizette Monteith, pharmaceutical services manager with Kheth’Impilo.

“One of the key objectives is job creation by addressing the skills gap in the South African economy,” Monteith says.

“One of the key objectives is job creation by addressing the skills gap in the South African economy.”

Lizette Monteith

The project has expanded from a handful of students in 2011 to more than 800 last year. This training programme is one of two task-shifting pharmaceutical-care models that have been developed to address the shortage of pharmacists in South Africa. The other trains nurses to dispense medicines.

A study published in April 2016 in the *Journal of Acquired Immune Deficiency Syndrome* comparing the two approaches found that the model using indirectly supervised pharmacist assistants provided a higher quality of pharmaceutical care and was less costly to implement than the nurse-managed model.

“We have seen a reduction in expired medication and fewer stock-outs at facilities employing pharmacist assistants. These are great results, but we need more analysis because we are looking at different models and different populations in this study,” says Monteith, one of the authors.

The assistants, many of whom were previously unemployed, are reaping social and economic benefits too. “More than 400 people have completed the two-year training and many of them were unemployed. Now they are bringing more than 62 million rand (US$ 4.4 million) a year in salaries back home and into their communities,” says Monteith.

“The students are incredibly dedicated. One group of trainees drove about 350 km, from their homes in the Eastern Cape to the town of East London, for classes every week for a whole year. They were never late and they never left early,” Monteith says.

Another Social Innovation in Health Initiative project is the Learner Treatment Kit project in schools in Malawi, which is run by Save the Children in partnership with the health and education ministries.

Teachers participating in the project provide malaria testing and treatment to their pupils at school. The service reduces absenteeism because children come to school even when they feel unwell, knowing they can get diagnosis and treatment there.

It adds to the teachers’ workload, but the teachers are motivated to provide the service because of the wider benefits, according to Victor Kadzinje, who co-ordinates the project.

“The learner treatment kit is the only medical health service readily available in primary schools during core school hours. It is a life-saving service,” Kadzinje says.

**Figure:**
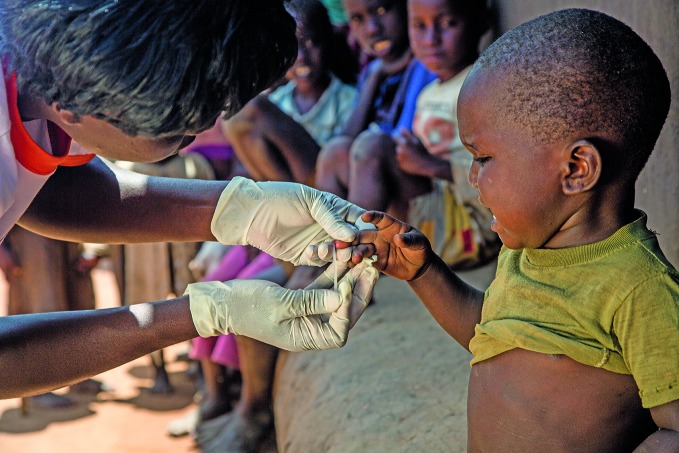
A Living Goods community health worker tests a child for malaria with a rapid diagnostic test in Busia County, western Kenya.

Living Goods

**Figure:**
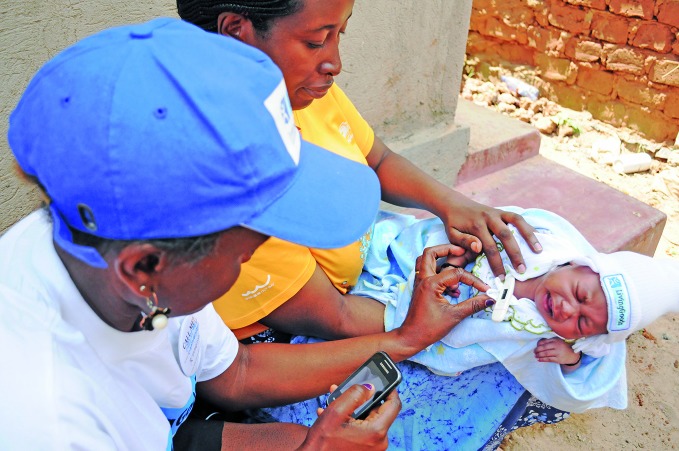
In Uganda, a Living Goods community health worker records the temperature of a newborn baby, using an app on her mobile phone.

Living Goods/Tine Frank

